# Sex differences in predictors and outcomes of camouflaging: Comparing diagnosed autistic, high autistic trait and low autistic trait young adults

**DOI:** 10.1177/13623613221098240

**Published:** 2022-06-02

**Authors:** Victoria Milner, Will Mandy, Francesca Happé, Emma Colvert

**Affiliations:** 1 King’s College London, UK; 2 University College London, UK

**Keywords:** autism, camouflaging, compensation, female autism, sex differences in autism

## Abstract

Many autistic individuals camouflage socially atypical behaviours. Evidence suggests autistic females camouflage more than autistic males. Although camouflaging may confer some benefits, it is also associated with negative outcomes including poorer mental health and well-being. Those with high autistic traits but no clinical diagnosis are not seldom included in camouflaging research, therefore we cannot ascertain whether camouflaging plays a role in the underdiagnosis of autistic females. Data from young adults with a diagnosis of autism (*n* = 78), high autistic traits but no diagnosis (*n* = 177) or low autistic traits (*n* = 180) revealed autistic females reported camouflaging significantly more than other groups. Males and females with low autistic traits reported significantly lower camouflaging than high trait and diagnosed groups. Loneliness was a key predictor of camouflaging for the diagnosed group only. Camouflaging was found to predict lower psychological quality of life for the diagnosed group, and lower social quality of life for the high trait and low trait groups. Overall, findings indicated that, although all groups reported camouflaging, the motivations for doing so may be different for diagnosed autistic individuals. It is important for stakeholders and society to improve understanding of autism and acceptance of atypical behaviour to alleviate possible negative outcomes associated with camouflaging.

**Lay Abstract:**

Many autistic people use strategies that help them adapt in social situations and hide behaviours that may seem different to non-autistic individuals – this is called camouflaging. Camouflaging may help autistic people fit in socially; however, it might also lead to poorer well-being. It has been suggested that autistic females camouflage more than autistic males. This article explored differences between males and females who have an autism diagnosis, have characteristics of autism but no diagnosis and those with few autistic characteristics. It is important to include these groups as camouflaging may make it more difficult to get an autism diagnosis and therefore make it less likely a person will receive support. We found that autistic women camouflaged more than all other groups. The group with few autistic characteristics (males and females) camouflaged the least. Loneliness was found to be a possible reason for camouflaging for the diagnosed autistic group only. In terms of outcomes related to camouflaging, it was found that those who camouflaged most had a lower quality of life; this was true of all groups. This tells us that there may be different reasons to camouflage, and different outcomes related to camouflaging for those with many characteristics of autism (including those with a diagnosis), and those with few. It is important that clinicians, teachers, parents and other stakeholders are aware of the negative outcomes associated with camouflaging so that more support can be provided for those who need it.

In recent years, researchers have adopted the term ‘camouflaging’ to describe strategies used to disguise socially ‘atypical’ behaviours, allowing an individual to ‘fit in’ (Hull et al., 2020). A wide range of strategies fit under this umbrella term, from conscious and learned behaviours such as rehearsed prompts or scripts to use in conversations, to unconscious and implicit behaviours such as mimicking the facial expressions of someone you are conversing with (See Cook et al., 2021 for a detailed review of camouflaging strategies). These strategies may be implemented, to some extent, by the majority of individuals for example, one might adapt ways of speaking to an employer or grandparent in comparison to a friend. Despite this, the use of camouflaging behaviours has been largely explored within the context of the autistic population. Evidence has consistently shown that autistic individuals report camouflaging more than non-autistic peers (Hull et al., 2019). However, not all autistic people report engaging in camouflaging behaviour, and some evidence suggests that factors, such as sex, gender and degree of autistic traits, contribute to differences in the use of these strategies (Cook et al., 2021). Please note that the authors use identity-first terminology throughout this report, in line with preferences reported by the autism community, while recognising that some individuals prefer person-first terminology (Kenny et al., 2016).

Camouflaging behaviour has been put forward as one potential explanation for the current male preponderance in diagnosed autism, with the male-to-female ratio estimated to be 3-to-1 (Loomes et al., 2017). The ‘camouflaging hypothesis’ posits that autistic females are more likely to successfully adopt camouflaging strategies than autistic males, disguising their social and communication difficulties. In line with this, it has been suggested that culture-based gender role expectations may lead autistic females to modify their behaviour to adopt more intrapersonal processes (e.g. mimicry of gender normative behaviour) more so than males (Kreiser & White, 2014). Such strategies impact the likelihood that autism-related difficulties will be recognised by relatives, teachers and health professionals and therefore restricts access to diagnostic pathways (Gould & Ashton-Smith, 2011). In line with this suggestion, a recent systematic review of 20 peer-reviewed papers found that camouflaging may be a major barrier to diagnosis for young women and girls (Lockwood-Estrin et al., 2021).

A considerable body of qualitative research supports the notion that camouflaging impacts access to diagnostic pathways for autistic females. For example, Leedham et al. (2020) interviewed 11 autistic women diagnosed after the age of 40 years who reported adopting camouflaging behaviours prior to diagnosis. The late-diagnosed women reflected that camouflaging pre-diagnosis may have contributed to unrecognised and unmet needs (Leedham et al., 2020). This was also found by Bargiela et al. (2016) who spoke to 14 autistic women and found that these women, who had all received a diagnosis in late adolescence or adulthood, reported camouflaging their autistic behaviours, which was implicated in poor recognition of their need for help. Similar findings were reported by Hull et al. (2017), who interviewed 55 autistic females, 30 autistic males and 7 autistic people who identified as a different gender. Participants in this study commented that females often ‘pretend to be normal’ and therefore are often overlooked for autism diagnosis. Interestingly, in an interview study conducted by Milner et al. (2019), participants suggested that autistic males experience less pressure to camouflage their autistic behaviours than autistic females, supporting the idea that societal gender role expectations may play a role in the amount of camouflaging seen. However, it should be noted that this sample consisted solely of females (autistic women and mothers of autistic girls).

Quantitative research has also explored the potential sex differences in camouflaging. Lai et al. (2017) used a discrepancy approach to investigate camouflaging behaviour whereby the researchers examined the differences in scores from observer measures (the Autism Diagnostic Observation Schedule (ADOS)) and self-report measures (the Autism Quotient (AQ), and the Reading the Mind in the Eyes Test). Using this method, they found that despite autistic females and males reporting similar AQ and mentalising scores, autistic females demonstrated fewer autistic traits during the observation measure, suggesting more camouflaging of traits for the females. Other studies adopting observation methods have found that autistic females demonstrate greater non-verbal social skills (Rynkiewicz et al., 2016), more social behaviours (Dean et al., 2017) and increased social language (Parish-Morris et al., 2017) compared to autistic males.

Hull et al. (2019) developed the first quantitative self-report measure of camouflaging, the Camouflaging Autistic Traits Questionnaire (CAT-Q). This measure captures three subdomains of camouflaging: compensation, masking and assimilation. Compensation refers to strategies that reduce the presentation of ‘atypical behaviours’ despite underlying difficulties existing, for example, learning and rehearsing a social script or prompts. Masking refers to the suppression of autistic behaviours, such as avoiding talking about a particular topic at length or learning to look at someone between the eyes to give the appearance of eye contact. Assimilation refers to adopting behaviours or interests to fit in with peers or a social group, for example, copying body language, clothing or interests. Using the CAT-Q, researchers found autistic females scored significantly higher than autistic males (Hull et al., 2019); however, these sex differences have not been replicated across all studies using the CAT-Q (e.g. Cage & Troxell-Whitman, 2019).

A major limitation of the majority of the research into camouflaging thus far is that samples have primarily included only clinically diagnosed autistic individuals, and a low-autistic trait comparison group. This poses a risk of circularity; if an individual successfully camouflages or compensates for their autistic behaviours, they are less likely to receive a diagnosis and would therefore be excluded from the clinical samples often used in this field of research. It is likely, therefore, that the true impact of camouflaging is not yet fully understood. Wood-Downie et al. (2021) attempted to address this concern, by including children and adolescents who scored above threshold on measures of autistic traits, but did not have a clinical diagnosis, within their ‘autism’ participant group. Using both discrepancy and observational methods, the researchers found that autistic girls (including those with high autistic traits but undiagnosed) demonstrated camouflaging, whereas autistic boys did not. However, by merging those with high autistic traits but no diagnosis and diagnosed autistic individuals into the same participant group, it is still unclear whether there are disparities in the rate of camouflaging between the two. It could be, for example, that if camouflaging reduces the likelihood of diagnosis, individuals with ‘high autistic traits’ but no diagnosis may have significantly higher camouflaging scores than those who have obtained a diagnosis.

In addition to examining the prevalence of camouflaging behaviour in the autistic population, recent research has also begun to examine the motivations and consequences of these processes. In terms of possible motivations or predictors for camouflaging behaviour, research has shown both conventional (such as success in the workplace) and relational (such as fitting in with friends) factors may be the driving force (Cage & Troxell-Whitman, 2019). Similarly, Hull et al. (2017) found motivations for camouflaging reported by late-diagnosed autistic women included assimilation and a desire to build relationships. Sex differences have been observed in terms of the motivations for camouflaging, with females citing conventional factors more often than males. Furthermore, a systematic review concluded that increased loneliness and a desire to fit in may be particularly strong motivators for camouflaging for autistic females (Tubío-Fungueiriño et al., 2021).

Although there may be benefits of adopting camouflaging strategies, such as greater success in social encounters, there are reports that consistently suggest camouflaging can be detrimental to well-being (e.g. Cook et al., 2021). There is evidence to suggest that camouflaging behaviour is associated with increased self-reported stress and anxiety (Cage & Troxell-Whitman, 2019; Hull et al., 2017). Camouflaging behaviour has also been linked to increased exhaustion for those engaging in this often-effortful strategy (Bargiela et al., 2016). Bargiela et al.’s (2016) qualitative examination also found that some individuals who adopted camouflaging techniques felt a loss of their sense of identity. Comparisons of autistic males and females have been mixed, with some studies reporting greater negative consequences for females (Lai et al., 2017) and others suggesting similar levels of negative consequences regardless of sex (Cage & Troxell-Whitman, 2019; Hull et al., 2019). Beck et al. (2020) found that in a group of women with high autistic traits (but no formal diagnosis of autism), higher levels of camouflaging significantly predicted higher levels of psychological distress. Furthermore, Tint and Weiss’ (2018) qualitative study with diagnosed autistic women suggested that camouflaging behaviours, specifically masking, may contribute to misunderstanding of autistic women’s clinical needs, potentially leading to further difficulties for this group. Perhaps most worryingly, recent research has indicated that camouflaging behaviour is a risk marker for suicidality in autistic adults (Cassidy et al., 2018), with this being a particularly pertinent factor for females (Beck et al., 2020).

This study aims to examine camouflaging and its possible predictors and consequences from data extracted from two cross-sectional studies: The Social Relationships Study (SR Study) and the Gender Differences in Social Communication (GDISC) study. Both the SR and GDISC studies included participants spanning the full autism spectrum and to address possible issues of circularity seen in previous research, both studies included diagnosed and non-diagnosed high autistic trait individuals. In all, the studies allowed examination of camouflaging behaviour in three distinct groups, those with a formal diagnosis of autism, high trait non-diagnosed individuals and a non-autistic comparison group (selected to be low in autistic trait levels). For all groups, both males and females took part in the study to examine sex differences. In addition to measures of camouflaging (the CAT-Q) and autistic traits, the studies included a battery of measures of well-being and mental health. Measures of happiness, loneliness and quality of life were used to examine possible motivations for/consequences of adopting camouflaging behaviours; these were selected from the available study measures based on previous evidence suggesting camouflaging may be associated with myriad aspects related to mental health and well-being.

This study aimed to compare CAT-Q camouflaging scores between high autistic trait (HT), diagnosed autistic (Dx) and comparison groups (CO), exploring group and sex differences. We predicted the following:

1. Females will have higher scores on the CAT-Q than males, in each diagnostic group (i.e. Dx, HT and CO).
2. There will be a positive correlation between CAT-Q scores and autistic traits.
3. Predictors of camouflaging will vary by group and sex.
4. Camouflaging will predict more negative outcomes (e.g. happiness and quality of life) for females than males in each group.

## Method

### Participants

Each group included participants from both the SR and GDISC studies. The SR Study is one of the largest population-based, longitudinal twin studies of the full autism spectrum. The SR Study sample was initially drawn from the Twins Early Development (TEDS) population (see Haworth et al., 2013 for details) and aimed to include all families in which one or both twins were suspected or confirmed to meet criteria for an autism diagnosis. Full details of the sample ascertainment can be found in Colvert et al. (2015). Data included in this article were collected during the SR Study’s third phase, which examined sex differences across a range of domains. The GDISC study is an online questionnaire-based study, which aimed to compare factors associated with social communication abilities between males and females in both autistic and non-autistic samples. Participants aged 20–25 years were invited to participate in the GDISC study between 2017 and 2019. This age bracket and time frame coincide with the age of the SR Study participants.

The diagnosed (Dx) group all had a formal clinical diagnosis of autism, this information was gathered across the three phases of the SR Study and for the GDISC participants the presence of a diagnosis was reported as part of the online questionnaires forming the study (in all *N* = 12; *n* = 3 males, *n* = 9 females reported a formal clinical diagnosis in GDISC). The high trait (HT) group were derived in two ways, those from the SR Study consisted of individuals scoring above cut-off on the Childhood Autism Spectrum Test (CAST; Scott et al., 2002) at age 8 or 12 years, and/or having current scores (when assessed at age 20–25) above 60 on the Social Responsiveness Scale – Second Edition (SRS-2; Constantino & Gruber, 2012). The GDISC questionnaire also included the SRS-2 to assess levels of autism traits. In all, *N* = 105 (*n* = 52 males, *n* = 53 females) in GDISC had high autism traits, but no formal diagnosis, and were therefore categorised in the relevant HT groups. The comparison sample consisted of the non-autistic co-twins of the SR Study Dx and HT twins, and any remaining individuals recruited via GDISC who had neither a clinical autism diagnosis nor high autism traits. Participants’ mean age and age range can be found in Table 1; there were no significant group effects on age. Demographic information regarding cognitive ability, ethnicity and socio-economic status were not available.

**Table 1.** Participant demographic information.

Sample group	Mean age	Age range
Dx males *N* = 35	22.52	20.78–24.84
Dx females *N* = 43	22.58	18.43–25.78
HT males *N* = 89	22.49	20.55–25.04
HT females *N* = 88	22.42	20.48–26.02
Comparison males *N* = 49	22.14	20.11–25.66
Comparison females *N* = 131	22.34	19.66–28.12

Dx: diagnosed group; HT: high trait group.

NB: For all groups sex is based on biological sex reported by parents in their first contact with the TEDS study or via the GDISC online questionnaire.

### Measures

#### Camouflaging Autistic Traits Questionnaire (CAT-Q)

To assess camouflaging behaviour, all participants completed the CAT-Q (Hull et al., 2019). At the time of the current research, the CAT-Q was still in development and as such a 32-item version of the questionnaire was used as opposed to the 25-item version used subsequently. For all questions, participants responded using a 7-point Likert-type scale ranging from 1 (*strongly disagree*) to 7 (*strongly agree*) for all statements. A sum of all items was calculated and is used in analyses. Higher scores indicated more camouflaging strategies used.

#### Social Responsiveness Scale (Second Edition) (SRS-2)

Autism traits were assessed using the SRS-2, a 65-item questionnaire assessing social ability (Constantino & Gruber, 2012). Each item on the SRS-2 is rated on a 4-point Likert-type scale from 1 (*not true*) to 4 (*almost always true*). For this study, the cut-off of 60 and above was set to determine high levels of autism traits, and this was used for the categorisation of sample groups only; for analyses, continuous raw scores are used.

#### Friendship Quality Scale (FQS)

The 23-item FQS was used as a measure of friendship relationships in this study (Bukowski et al., 1994). The scale is designed to assess relationships with a best friend and yields scores for five aspects of friendship: companionship, conflict, help, security and closeness. For this study, only the overall mean score is included in analyses to limit the number of statistical comparisons and lower the risk of type 1 error.

#### UCLA loneliness scale – shortened version

To assess loneliness as a possible predictor and/or consequence of camouflaging, a shortened version of the UCLA loneliness scale was used (Russell, 1996). Four items from version 3 of the UCLA scale were selected; ‘How often do you feel alone?’; ‘how often do you feel left out?’; ‘how often do you feel isolated from others?’; and ‘how often do you feel that people are around you but not with you?’. Two study team devised questions were added to the measure based on qualitative findings (Milner et al., 2019); ‘how often do you feel lonely when you are on your own?’; and ‘how often do you like being on your own?’ (this last question was reverse coded for analyses). All questions were scored on a 5-point scale ranging from 1 (*never*) to 5 (*always*). For analyses, responses were summed to create a total loneliness score.

#### Subjective happiness scale

Happiness was assessed using the 4-item subjective happiness scale (Lyubomirsky & Lepper, 1999). Each item required the participant to choose from seven response options to finish a sentence fragment, for example, ‘In general I consider myself . . .’ with choices ranging from 1 (*not a very happy person*) to 7 (*a very happy person*). A mean of all four items was used for analyses, with higher scores indicating higher levels of happiness. This measure was only completed by participants in the SR Study, as it formed part of the task battery for the main research project and as such results are confined to the Dx and HT groups.

#### World Health Organization Quality of Life Scale (WHO-QoL Bref)

Quality of life was assessed using the WHO-QoL Bref, a 26-item questionnaire designed by the World Health Organization to assess four domains: physical quality of life, psychological quality of life, social relationships and environmental quality of life (The WHOQOL Group, 1998).

#### Strengths and Difficulties Questionnaire (SDQ)

Emotional and behavioural difficulties were measured using the informant-reported SDQ (Goodman, 1997). This measure was only completed by those participants in the SR Study, as it formed part of the task battery for the main research project and as such results are confined to just the Dx and HT groups. Each item on the SDQ is scored 0 (*does not apply*), 1 (*applies somewhat*) or 2 (*certainly applies*). Composite scores were calculated for five separate domains: emotional difficulties, conduct problems, hyperactivity, peer problems and pro-social behaviour, with emotional and peer being examined as possible consequences of camouflaging.

### Data analysis

Data from both studies were combined, organised and analysed using IBM SPSS Statistics version 27. Chi-square analysis was conducted to compare those with missing and complete data; these analyses revealed that there were no significant differences in the sex of participants (i.e. males were no more likely to not complete the data than females). Participants in the comparison group were more likely to have incomplete/missing data than the HT or Dx groups. However, due to the nature of online surveys, and comparable sample size compared with HT and Dx groups, all participants with incomplete/missing data were excluded from further analysis (*n* = 84 removed).

To address Hypothesis 1, a 2 (sex: male, female) × 3 (diagnostic group: Dx, HT, CO) ANOVA was conducted to explore group differences in total CAT-Q score.

To address Hypothesis 2, correlation analysis was conducted to determine whether there was a significant relationship between CAT-Q scores (camouflaging) and SRS-2 scores (autistic traits). Because SRS-2 scores were not normally distributed and some violated assumptions of linearity, Spearman-rank correlations were used to test this hypothesis. This analysis was conducted for each of the diagnostic groups split by sex (i.e. Dx males, Dx females, HT males, HT females, CO males and CO females). To assess group differences in the strength of correlation between camouflaging and autistic traits, Fisher’s r-z transformations were computed (using an online calculator http://onlinestatbook.com/2/calculators/r_to_z.html).

To address Hypothesis 3, based on the literature, out of the variables in this study loneliness was deemed to be the most likely predictor of camouflaging. Therefore, to explore this, a three-step hierarchical regression model was run for each diagnostic group (Dx, HT, CO) to determine whether sex and/or loneliness or the interaction of these variables predicted camouflaging scores. To control for level of autistic traits, SRS-2 scores were included in the model (Block 1). To examine within-group sex differences, main effect of sex (Block 2) and the interaction between sex and loneliness (Block 3) were also included in the regression model.

To address Hypothesis 4, to examine potential outcomes related to camouflaging, a series of three-step hierarchical regressions were carried out within each diagnostic group (Dx, HT, comparison) separately. To determine whether camouflaging scores statistically predict outcomes over and above sex and level of autistic traits, the first block of variables in each regression model included SRS-2 score and sex. The second block included CAT-Q total score. The third block included an interaction term of sex × CAT-Q total.

## Results

Although the Dx and HT participants are taken from a twin sample, for the present analyses, they were treated as singletons (i.e. each member of the twin pairs is included in analyses). This is due to the comparison group being drawn from a non-twin population and also to maximise the power to compare the Dx, HT and comparison samples. Table 2 shows the mean scores and standard deviations for each variable split by group and sex.

**Table 2.** Means and standard deviations for predictor and predicted variables split by participant group and sex.

Sample group	Total CAT-Q		SRS-2		Loneliness		FQS		Subjective happiness		WHO-QoL physical		WHO-QoL psychological		WHO-QoL social		WHO-QoL environmental		SDQ emotional		SDQ peer	
	*N*	Mean (*SD*)	*N*	Mean (*SD*)	*N*	Mean (*SD*)	*N*	Mean (*SD*)	*N*	Mean (*SD*)	*N*	Mean (*SD*)	*N*	Mean (*SD*)	*N*	Mean (*SD*)	*N*	Mean (*SD*)	*N*	Mean (*SD*)	*N*	Mean (*SD*)
Dx males	34	97.35 (22.27)	34	77.68(24.8)	34	16.47 (4.67)	33	3.72 (0.54)	30	3.76 (1.5)	32	15.05 (2.47)	32	12.13 (2.85)	33	12.71 (3.29)	32	14.58 (2.03)	11	4.00 (2.32)	11	3.27 (2.05)
Dx females	42	108.76 (24.33)	41	100.85 (25.25)	43	17.37 (5.41)	43	3.91 (0.57)	32	4.04 (1.52)	40	13.97 (2.70)	41	11.46 (3.07)	39	13.40 (3.77)	41	14.10 (2.75)	11	6.00 (2.61)	11	4.09 (1.97)
HT males	89	103.51 (18.93)	89	80.82 (17.15)	89	16.06 (4.10)	89	3.89 (0.48)	34	4.13 (1.32)	86	15.06 (2.34)	86	12.60 (3.07)	86	12.82 (3.52)	86	14.63 (2.58)	15	1.33 (1.88)	15	.60 (0.83)
HT females	87	100.01 (25.49)	87	78.77 (21.35)	87	15.74 (4.14)	87	3.00 (0.47)	34	3.87 (1.36)	86	14.93 (2.34)	86	12.30 (2.60)	86	13.29 (2.91)	86	14.69 (2.03)	15	3.60 (2.82)	15	2.20 (2.27)
Comparison males	41	79.63 (18.28)	43	43.60 (16.33)	46	13.87 (4.82)	46	3.89 (0.54)			40	16.33 (2.10)	39	14.56 (3.11)	40	14.80 (3.30)	40	15.53 (2.54)				
Comparison females	105	77.94 (21.01)	100	37.33 (13.31)	107	14.21 (2.68)	107	4.26 (0.42)			92	16.30 (2.07)	92	13.84 (2.55)	92	15.45 (3.02)	92	15.86 (1.89)				
Group × Sex ANOVA	*F*_(5)_ = 23.51, *p* < 0.001		*F*_(5)_ = 110 33, *p* < 0.001		*F*_(5)_ = 5.97, *p* < 0.001		*F*_(5)_ = 10.46, *p* < 0.001		*F*_(3)_ = 0.43, *p* = 0.73		*F*_(5)_ = 8.60, *p* < 0.001		*F*_(5)_ = 8.16, *p* < 0.001		*F*_(5)_ = 7.99, *p* < 0.001		*F*_(5)_ = 5.59, *p* < 0.001		*F*_(3)_ = 8.03, *p* < 0.001		*F*_(3)_ = 8.73, *p* < 0.001	
ANOVA Post Hoc	Dx > CO HT > CO DxF > DxM		Dx > CO Dx > HT HT > CO DxF > DxM		Dx > CO HT > CO DxF > COF HTF > COF DxM > COM HTM > COM		Dx < CO HT < CO *F* > M DxF > COF HTF > COF COF > COM		n/a		Dx < CO HT < CO DxF > DxM DxF > COF HTF > COF DxM > COM HTM > COM		Dx < CO HT < CO DxF > COF HTF > COF DxM > COM HTM > COM		Dx < CO HT < CO DxF > COF HTF > COF DxM > COM HTM > COM		Dx < CO, HT < CO DxF > COF HTF > COF		Dx > HT *F* > M DxF > HTF DxM > HTM DxF > DxM HTF > HTM		Dx > HT *F* > M DxF > HTF DxM > HTM HTF > HTM	

CAT-Q: Camouflaging Autistic Traits Questionnaire; SRS-2: Social Responsiveness Scale – Second Edition; FQS: Friendship Quality Scale; WHO-QoL: World Health Organization Quality of Life Measure; SDQ: Strengths and Difficulties Questionnaire; Dx: Diagnosed Group; HT: High Trait Group, CO: Comparison Group, F: female, M: male.

### Hypothesis 1 – females will have higher scores on the CAT-Q in all diagnostic groups (i.e. Dx, HT and CO)

There was no significant main effect of sex, *F*(1, 392) = 0.738, *p* = 0.391, partial η^2^ = 0.002. There was a statistically significant main effect of group, *F*(2,392) = 45.91, *p* < 0.0001, partial η^2^ = 0.19. Post hoc analysis revealed that for both males and females, the comparison groups showed significantly lower scores on the CAT-Q than both the Dx and the HT groups (all *p*s < 0.001).

In addition, there was a statistically significant interaction between sex and group, *F*(2,392) = 3.21, *p* = 0.042, partial η^2^ = 0.016. Simple main effects analysis revealed that for the Dx group, CAT-Q total scores were significantly higher for females than males, with a mean difference of 11.41, 95% CI [1.49, 21.32], *F*(1,392) = 5.12, *p* = 0.024, partial η^2^ = 0.013. Sex differences were not significant for either HT or comparison groups.

### Hypothesis 2 – there will be a positive correlation between CAT-Q scores and autistic traits

Correlation analyses were conducted to assess the relationship between camouflaging (CAT-Q total score) and autistic traits (SRS-2 score) for each group, split by sex. The outcomes of these analyses are shown in Table 3. In summary, there were statistically significant associations between CAT-Q total and SRS-2 scores for all groups except for Dx males and CO males (*p* > 0.05).

**Table 3.** Correlations between CAT-Q total and SRS-2 scores split by group and sex.

	Female		Male		Total	
	*n*	*r_s_*	*n*	*r_s_*	*n*	*r_s_*
Dx	42	0.377*	34	0.208	76	0.359***
HT	87	0.481***	89	0.369***	176	0.436***
Comp	99	0.433***	40	0.265	151	0.405***

Dx: Diagnosed Group; HT: high trait group; Comp: comparison group; *r_s_*: Spearman’s rho.

* *p* < 0.05, ***p < 0.001.

Fischer’s *r*-*z* calculations revealed no significant differences in the strength of correlation when comparing within-group sex differences (e.g. Dx females vs Dx male), within sex group differences (e.g. Dx male vs HT male) or group differences (e.g. Dx vs HT) (all *p*s > 0.05).

### Hypothesis 3 – predictors of camouflaging will vary by group and sex

Although the final models were significant for all groups, loneliness was a significant predictor of camouflaging for the Dx group only. The effect of sex and the interaction between sex and loneliness were not significant for any group. Details of the regression outcomes can be found in Table 4.

**Table 4.** Loneliness as a predictor of camouflaging for each participant group.

Group	Predictor	Unstandardized coefficient	*t*	*p*-value	*F*	*R* ^2^
DX						
Block 3:	SRS-2 score	0.11	1.03	0.308		
	Sex	19.67	1.06	0.295		
	**Loneliness**	**2.04**	**3.12**	**0.003**		
	Sex × Loneliness	−1.62	−1.56	0.123	4.78**	0.214
HT						
Block 3:	**SRS-2 score**	**0.441**	**5.43**	**0.000**		
	Sex	−4.67	−0.39	0.694		
	Loneliness	0.969	1.85	0.066		
	Sex × Loneliness	0.433	0.60	0.549	14.53***	0.254
Comparison						
Block 3:	**SRS-2 score**	**0.522**	**4.2 9**	**0.000**		
	Sex	−15.03	−1.07	0.288		
	Loneliness	0.988	1.34	0.183		
	Sex × Loneliness	1.11	1.13	0.262	9.60***	0.223

DX: diagnosed group; HT: high trait group; Comp: comparison group. Bold denotes predictor significant at *p* < 0.05.

* *p* < 0.05, ***p < 0.001.

### Hypothesis 4 – camouflaging will predict more negative outcomes for females than males in each group

Tables 5 to 7 summarise the final model of the regression outputs for the diagnosed, high trait and comparison groups, respectively. Details of the total regression model can be found in the supplementary materials. Regression analyses are only reported for outcome variables that significantly correlated with CAT-Q total score and therefore number of outcomes reported vary per group.

**Table 5.** Prediction of Outcomes from Camouflaging, Sex, and Their Interaction for Diagnosed Autistic (Dx) participants.

Outcome and model	Predictor	Unstandardized coefficient	*t*	*p*-value	Sig. after correction	*F*	*R* ^2^
Subjective happiness (*n* = 62)							
Block 3:	**SRS-2 score**	−**0.03**	−**3.52**	**0.001**	*		
	Sex	−1.33	−0.89	0.377	–		
	CAT-Q total	−0.02	−1.59	0.117	–		
	CAT-Q × Sex	0.00	0.246	0.806	–	5.33***	0.272
Psychological QoL (*n* = 73)							
Block 3:	**SRS-2 score**	−**0.06**	−**4.87**	**0.000**	*		
	Sex	−3.97	−1.46	0.148	–		
	**CAT-Q total**	−**0.04**	−**2.60**	**0.011**	*		
	CAT-Q × Sex	0.03	1.07	0.289	–	10.19***	0.375
SDQ emotional subscale (*n* = 22)							
Block 3:	SRS-2 score	0.04	1.42	0.174	–		
	Sex	6.05	1.40	0.179	–		
	**CAT-Q total**	**0.06**	**2.24**	**0.039**	*		
	CAT-Q × Sex	−0.063	−1.69	0.110	–	2.95	0.410

QoL: quality of life; SDQ: Strengths and Difficulties Questionnaire; Sig.: significance.

Bold denotes predictor significant at *p* < 0.05. For Sig. After Correction × Predictor significant after Bonferroni correction for multiple comparisons. For *F* and *R*^2^: *p < 0.05, **p < 0.01, ***p < 0.001.

**Table 6.** Prediction of Outcomes from Camouflaging, Sex and Their Interaction for High Autistic Trait (HT) participants.

Outcome and model	Predictor	Unstandardized coefficient	*t*	*p*-value	Sig. after correction	*F*	*R* ^2^
Physical QoL (*n* = 172)							
Block 3:	**SRS-2 score**	−**0.044**	−**4.45**	**0.000**	*		
	Sex	2.26	1.39	0.167	–		
	CAT-Q total	0.00	0.43	0.667	–		
	CAT-Q × Sex	−0.02	−1.28	0.202	–	6.99***	0.143
Psychological QoL (*n* = 172)							
Block 3:	**SRS-2 score**	−**0.05**	−**4.15**	**0.000**	*		
	Sex	2.58	1.32	0.189	–		
	CAT-Q total	−0.01	−0.65	0.514	–		
	CAT-Q × Sex	−0.021	−1.12	0.266	–	8.45***	0.168
Social QoL (*n* = 172)							
Block 3:	SRS-2 score	−0.02	−1.31	0.190	–		
	Sex	4.37	1.88	0.062	–		
	CAT-Q total	−0.01	−0.683	0.495	–		
	**CAT-Q** × **Sex**	−**0.046**	−**2.07**	**0.040**	–	4.17**	0.091*
Environmental QoL (*n* = 172)							
Block 3:	**SRS-2 score**	−**0.03**	−**2.67**	**0.008**	–		
	Sex	1.95	1.16	0.248	–		
	CAT-Q total	0.000	−0.04	0.966	–		
	CAT-Q × Sex	−0.019	−1.17	0.243	–	3.36**	0.075
SDQ Emotional Subscale (*n* = 30)							
Block 3:	SRS-2 score	−0.02	−0.665	0.512	–		
	Sex	−5.10	−1.08	0.291	–		
	CAT-Q total	0.00	0.16	0.873	–		
	CAT-Q × Sex	0.028	0.626	0.537	–	1.92	0.235
SDQ Peer Subscale (*n* = 30)							
Block 3:	**SRS-2 score**	**0.05**	**2.93**	**0.007**	*		
	Sex	−2.40	−0.90	0.375	–		
	CAT-Q total	0.01	0.93	0.362	–		
	CAT-Q × Sex	0.01	0.35	0.728	–	6.80***	0.521

QoL: quality of life; SDQ: Strengths and Difficulties Questionnaire; Sig.: significance.

Bold denotes predictor significant at *p* < 0.05. For Sig. After Correction × Predictor significant after Bonferroni correction for multiple comparisons. For *F* and *R*^2^: *p < 0.05, **p < 0.01, ***p < 0.001.

**Table 7.** Prediction of Outcomes from Camouflaging, Sex, and Their Interaction for comparison (not autistic) participants.

Outcome and Model	Predictor	Unstandardized coefficient	*t*	*p*-value	Sig. after correction	*F*	*R* ^2^
FQS (*n* = 139)							
Block 3:	**SRS-2 score**	−**0.01**	−**3.45**	**0.001**	*		
	Sex	−0.21	−0.62	0.538	–		
	CAT-Q total	−0.00	−0.29	0.774	–		
	CAT-Q * Sex	−0.00	−0.37	0.714	–	9.58***	0.222
Physical QoL (*n* = 128)							
Block 3:	SRS-2 score	−0.03	−1.92	0.058	–		
	Sex	−0.85	−0.50	0.618	–		
	CAT-Q total	−0.01	−1.17	0.243	–		
	CAT-Q * Sex	0.013	0.650	0.517	–	2.00	0.061
Psychological QoL (*n* = 127)							
Block 3:	**SRS-2 score**	−**0.06**	−**3.05**	**0.003**	*		
	Sex	2.07	0.97	0.334	–		
	CAT-Q total	−0.02	−1.46	0.146	–		
	CAT-Q * Sex	−0.012	−0.468	0.640	–	6.01***	0.165
Social QoL (*n* = 128)							
Block 3:	**SRS-2 score**	−**.06**	−**2.92**	**0.004**	*		
	Sex	4.41	1.86	0.065	–		
	CAT-Q total	−0.02	−0.97	0.333	–		
	CAT-Q * Sex	−**0.063**	−**2.153**	**0.033**	–	7.38***	0.194*

FQS: Friendship Quality Scale; QoL: quality of life; Sig.: significance.

Bold denotes predictor significant at *p* < 0.05. For Sig. After Correction × Predictor significant after Bonferroni correction for multiple comparisons. For *F* and *R*^2^: *p < 0.05, **p < 0.01, ***p < 0.001.

For the diagnosed group, camouflaging significantly predicted lower psychological quality of life only. There were no significant sex × CAT-Q interactions.

For the high trait group, camouflaging significantly predicted lower social quality of life only. There was a significant sex × CAT-Q interaction for this outcome. Post hoc analyses revealed that CAT-Q total score significantly predicted lower social quality of life for high trait males *B* = −0.054, *p* = 0.008, but not for high trait females, *B* = 0.012, *p* = 0.402.

For the comparison group, camouflaging significantly predicted lower social quality of life only. Similar to the high trait group, there was a significant sex × CAT-Q interaction for this outcome. Post hoc analyses revealed that CAT-Q total score significantly predicted lower social quality of life for high trait males *B* = −0.083, *p* = 0.005, but not for high trait females, *B* = −0.012, *p* = 0.437.

## Discussion

This study aimed to investigate group and sex differences in camouflaging behaviour and the potential predictors and consequences of this. Taking the results as a whole, it was found that females with a diagnosis of autism endorsed camouflaging strategies more than males with a diagnosis. However, this sex difference was not present for those with high autistic traits but no diagnosis, nor for our comparison group. This pattern of findings partially supports our prediction for hypothesis one, as sex differences were apparent only for those with a clinical diagnosis. For both undiagnosed groups regardless of level of autistic traits, males and females reported the same level of camouflaging. Previous evidence has also demonstrated sex difference in clinical samples but not a non-autistic comparison group (Hull et al., 2020). As found in existing research, the non-autistic comparison group camouflaged significantly less than those with high levels of autistic traits and/or a clinical diagnosis (Hull et al., 2020).

Our second hypothesis predicted a relationship between levels of autistic traits and camouflaging and was supported for all female groups and the high trait males, but not for the diagnosed or comparison males. However, there were no significant differences in the strength of relationship between or within groups, therefore conclusions about sex differences in the relationship between camouflaging and autistic traits can only be tentative. These results may indicate that for females, there is a linear relationship indicating that higher levels of autistic traits may be related to higher levels of camouflaging; however, the pattern is potentially not so clear for males. This adds to the discussion of whether camouflaging is related solely to autistic traits, or whether additional factors are at play, particularly for males. Although further investigation is needed to confirm the possible differences in this relationship between camouflaging and autistic traits for males and females, one possible explanation for this finding is the role of culture and society. Expectations and acceptance of atypical social behaviours may vary depending on the sex of an individual. For example, in some cultures, a traditional view of the role of males and females may impact a person’s perceived need to fit in within social settings. Therefore, it could be that for females the more ‘atypical’ they feel, perhaps due to their autistic traits, the more they feel a pressure to camouflage. However, for males, it could be that their atypical behaviours are more readily accepted within society and perceived as strengths such as assertion or independence, rather than oddness, and thus camouflaging is less related to their level of autistic traits. In addition, the participants in this sample were all young adults. It is likely that cultural and society pressures evolve over time and across generations. Therefore, the influence of these pressures, including the disparity of pressure for males and females, may be different in a sample of older individuals. To fully address this possible interpretation, future research should explore the potential role of culture and society further.

We then explored our third hypothesis that the motivation for camouflaging, and therefore predictors, may vary between diagnostic groups and sexes. Our results revealed that loneliness was a significant statistical predictor of camouflaging for the diagnosed group only; however, there was no main effect or interaction with sex indicating that loneliness was equally a predictor of camouflaging for both males and females. For the high trait and comparison groups, the regression model demonstrated that camouflaging was driven by autistic traits (scores on the SRS-2) with no additional contribution from loneliness. Again, no sex differences were revealed. In contrast to the current study’s findings, previous research has found sex differences in predictors of camouflaging (Cage & Troxell-Whitman, 2019), with autistic females more focused on conventional type motivators (e.g. success at work) compared to autistic males. The group differences highlight that loneliness may play a unique role in the experience of camouflaging for autistic individuals. It has previously been shown that higher levels of autistic features are associated with increased loneliness (Schiltz et al., 2021), potentially due to non-autistic individuals’ lack of understanding and inclusion of those who are neurodivergent. Thus, it may be that some autistic individuals minimise autistic features via camouflaging with the aim of alleviating loneliness. There are likely many motivations for camouflaging, other than loneliness, which were not fully explored in this study. However, the results of this study add a novel contribution that although everyone may modulate their behaviour and interaction at some point in their lives, and arguably therefore camouflage, the motivation for doing so may be different for diagnosed autistic individuals.

Our final hypothesis explored potential negative outcomes related to camouflaging behaviour. We found that for the diagnosed participants, increased camouflaging predicted poorer psychological quality of life. The regression analysis indicated that this was the case for both diagnosed males and females. This is in line with evidence from Cage et al. (2018) who found camouflaging significantly predicted depression scores for both autistic males and females. For both the high trait and comparison groups, increased camouflaging predicted poorer social quality of life. Interestingly, for both these groups, there was a significant interaction with sex; the association of camouflaging with social quality of life was only significant for males but not females. Therefore, for the high trait and comparison females, camouflaging may lead to fewer negative outcomes – against our hypothesis. In both the high trait and comparison groups, males and females report camouflaging to a comparable extent, and show similar levels of social quality of life. Therefore, it is intriguing that camouflaging appears to only predict social quality of life for the males in these groups. Perhaps, camouflaging impacts other aspects of females’ lives which are not included in this study. Alternatively, this finding may reflect greater success when adopting camouflaging strategies for females in these groups. It is possible that for the high trait and comparison groups, societal expectations to camouflage or modulate social behaviours may be more reinforced in the females’ lives, therefore they are more effectively able to have their social needs met when adopting these strategies. However, this cannot be measured by the CAT-Q which only captures intent to use strategies, not the effectiveness when implemented.

It has previously been suggested that camouflaging may be a barrier to diagnosis for autistic females (Lockwood-Estrin et al., 2021), however we found no sex differences in camouflaging between our high trait males and females. Furthermore, our high trait group demonstrated similar levels of camouflaging to diagnosed males. Therefore, our findings do not support the notion that high trait females are being overlooked due to the extent to which they camouflage. As previously noted, the CAT-Q is a limited measure as it does not capture the subsequent success of strategies used. It could be that high trait females are more successful when using camouflaging, impacting identification and access to diagnostic pathways, but this cannot be concluded from the current findings. Future work could take a ‘discrepancy’ approach to measuring camouflaging, to test this possibility (e.g. Livingston et al., 2019). In the meantime, caution should be taken when making assumptions about sex differences in camouflaging and the potential impact on the male preponderance in autism.

Of all the sample groups in this study, the diagnosed autistic females were found to camouflage more than any other group, perhaps reflecting an acute awareness of autistic behaviours due to a diagnostic label and a subsequent desire to avoid stigma and discrimination. This may be more apparent for autistic females compared to males due to increased social motivation and desire to fit in with peers (Sedgewick et al., 2016). An alternative explanation may be that diagnosed autistic females received greater instruction on how to adapt their social behaviours from teachers and parents, compared to their male counterparts, reflecting increased societal demands on females. Interestingly, as this group of females have an existing diagnosis, camouflaging may not have impaired their access to clinical identification. However, we do not have information about age of diagnosis, onset of camouflaging and any additional difficulties which these individuals may have that potentially impacted their chances of diagnosis. Dworzynski et al. (2012), in a study using TEDS data from childhood, found that intellectual disability and/or behavioural (e.g. conduct disorder) issues differentiated high trait girls, but not boys, who did versus did not meet criteria for an autism diagnosis.

### Recommendations

From the findings of the current study, it is apparent that camouflaging strategies are frequently adopted by both males and females with high levels of autistic traits. Therefore, we recommend improved awareness, understanding and identification of camouflaging by key stakeholders including clinicians and teachers. This will potentially allow greater access to support for these individuals and may reduce some of the challenges in terms of accessing a clinical diagnosis.

In line with this, improvements should be made to increase the understanding and acceptance of autistic behaviours and remove stigma and discrimination related to these. This will reduce the perceived necessity to adopt camouflaging strategies which often leads individuals to disguise their authentic selves and may therefore impact the relationship seen between camouflaging and negative outcomes such as quality of life.

### Limitations

Although this study has many strengths such as including individuals with high autistic traits but no clinical diagnosis which avoids circularity found in some previous research, there are some limitations of the methods used. First, the study is cross-sectional in nature and therefore cannot determine the causal relationship between camouflaging and the other variables assessed. In addition, there is a disparity in sample sizes between groups which may have impacted the power to detect some group and sex differences. A binary definition of biological sex was adopted in this study, and future projects should consider gender identity and its relationship with camouflaging. Although the topic of this project stemmed from focus group data conducted with autistic individuals (Milner et al., 2019), there was no direct community involvement in the reported study. Importantly, although due to the nature of the online survey, it is likely that the majority of participants had average or above average cognitive ability, this was not formally measured or examined in this study. Previous studies have shown that aspects of cognition such as executive function influence the use of camouflaging strategies (Hull et al., 2021) and therefore should be explored further in relation to outcomes associated with camouflaging. Questions relating to mental health conditions were included on the original questionnaires; however, upon inspection of this data, the matrix table multiple endorsement format of the data collection was unreliable and misunderstood by some participants, as such these data were not deemed suitable for inclusion in the current manuscript. Furthermore, the majority of measures used in this study were self-report, and future studies should also examine objective measures of camouflaging, its predictors and associated outcomes. Finally, some participants, predominantly the comparison sample, were recruited via convenience sampling which may affect the representativeness of our findings.

### Future directions

Our current understanding of camouflaging autistic behaviours is in its infancy and, therefore, there are many possible areas for future research. First, other aspects of camouflaging strategies should be examined further, including the context and success of adopting these behaviours. There is currently also limited understanding regarding the developmental trajectory of camouflaging; for instance, when do individuals begin to adopt camouflaging strategies and is this similar for autistic males and females? It would be useful to expand our existing knowledge of predictors and outcomes associated with camouflaging, using longitudinal designs from an early age, as this has significant implications for the well-being of individuals who camouflage.

## Conclusion

In conclusion, the findings of this study suggest that diagnosed autistic males report least, and diagnosed autistic females report most camouflaging, with sex differences being less evident in undiagnosed high autism trait or comparison (low trait) groups. Levels of autistic traits are related to camouflaging behaviours for females, and for males with high or low levels of autistic traits but no autism diagnosis. Despite sex differences in the extent to which autistic individuals camouflage, there may be similar motivations and consequences of camouflaging. Interestingly, sex differences appear to exist for outcomes for individuals without a clinical diagnosis, which may reflect the efficacy of camouflaging strategies.

## Supplemental Material

sj-docx-1-aut-10.1177_13623613221098240 – Supplemental material for Sex differences in predictors and outcomes of camouflaging: Comparing diagnosed autistic, high autistic trait and low autistic trait young adults Click here for additional data file. Supplemental material, sj-docx-1-aut-10.1177_13623613221098240 for Sex differences in predictors and outcomes of camouflaging: Comparing diagnosed autistic, high autistic trait and low autistic trait young adults by Victoria Milner, Will Mandy, Francesca Happé and Emma Colvert in Autism

## References

[bibr1-13623613221098240] Bargiela S. Steward R. Mandy W. (2016). The experiences of late-diagnosed women with autism spectrum conditions: An investigation of the female autism phenotype. Journal of Autism and Developmental Disorders, 46(10), 3281–3294. 27457364 10.1007/s10803-016-2872-8 PMC5040731

[bibr2-13623613221098240] Beck J. S. Lundwall R. A. Gabrielsen T. Cox J. C. South M. (2020). Looking good but feeling bad: ‘Camouflaging’ behaviors and mental health in women with autistic traits. Autism, 24(4), 809–821. 32429817 10.1177/1362361320912147

[bibr3-13623613221098240] Bukowski W. M. Hoza B. Boivin M. (1994). Measuring friendship quality during pre-and early adolescence: The development and psychometric properties of the Friendship Qualities Scale. Journal of Social and Personal Relationships, 11(3), 471–484.

[bibr4-13623613221098240] Cage E. Di Monaco J. Newell V. (2018). Experiences of autism acceptance and mental health in autistic adults. Journal of Autism and Developmental Disorders, 48(2), 473–484. 29071566 10.1007/s10803-017-3342-7 PMC5807490

[bibr5-13623613221098240] Cage E. Troxell-Whitman Z. (2019). Understanding the reasons, contexts and costs of camouflaging for autistic adults. Journal of Autism and Developmental Disorders, 49(5), 1899–1911. 30627892 10.1007/s10803-018-03878-x PMC6483965

[bibr6-13623613221098240] Cassidy S. Bradley L. Shaw R. Baron-Cohen S. (2018). Risk markers for suicidality in autistic adults. Molecular Autism, 9(1), 1–14. 30083306 10.1186/s13229-018-0226-4 PMC6069847

[bibr7-13623613221098240] Colvert E. Tick B. McEwen F. Stewart C. Curran S. R. Woodhouse E. . . . Bolton P. (2015). Heritability of autism spectrum disorder in a UK population-based twin sample. Journal of the American Medical Association Psychiatry, 72, 415–423. 25738232 10.1001/jamapsychiatry.2014.3028 PMC4724890

[bibr8-13623613221098240] Constantino J. N. Gruber C. P. (2012). Social Responsiveness Scale: SRS-2. Western Psychological Services.

[bibr9-13623613221098240] Cook J. Hull L. Crane L. Mandy W. (2021). Camouflaging in autism: A systematic review. Clinical Psychology Review, 89, 102080. 34563942 10.1016/j.cpr.2021.102080

[bibr10-13623613221098240] Dean M. Harwood R. Kasari C. (2017). The art of camouflage: Gender differences in the social behaviors of girls and boys with autism spectrum disorder. Autism, 21(6), 678–689. 27899709 10.1177/1362361316671845

[bibr11-13623613221098240] Dworzynski K. Ronald A. Bolton P. Happé F. (2012). How different are girls and boys above and below the diagnostic threshold for autism spectrum disorders? Journal of the American Academy of Child & Adolescent Psychiatry, 51(8), 788–797. 22840550 10.1016/j.jaac.2012.05.018

[bibr12-13623613221098240] Goodman R. (1997). The strengths and difficulties questionnaire: A research note. Journal of Child Psychology and Psychiatry, 38, 581–586. 9255702 10.1111/j.1469-7610.1997.tb01545.x

[bibr13-13623613221098240] Gould J. Ashton-Smith J. (2011). Missed diagnosis or misdiagnosis? Girls and women on the autism spectrum. Good Autism Practice (GAP), 12(1), 34–41.

[bibr14-13623613221098240] Haworth C. M. Davis O. S. Plomin R. (2013). Twins Early Development Study (TEDS): A genetically sensitive investigation of cognitive and behavioural development from childhood to young adulthood. Twin Research & Human Genetics, 16, 117–125. 23110994 10.1017/thg.2012.91 PMC3817931

[bibr15-13623613221098240] Hull L. Mandy W. Lai M. C. Baron-Cohen S. Allison C. Smith P. Petrides K. V. (2019). Development and validation of the Camouflaging Autistic Traits Questionnaire (CAT-Q). Journal of Autism and Developmental Disorders, 49(3), 819–833. 30361940 10.1007/s10803-018-3792-6 PMC6394586

[bibr16-13623613221098240] Hull L. Petrides K. V. Allison C. Smith P. Baron-Cohen S. Lai M. C. Mandy W. (2017). ‘Putting on my best normal’: Social camouflaging in adults with autism spectrum conditions. Journal of Autism and Developmental Disorders, 47(8), 2519–2534. 28527095 10.1007/s10803-017-3166-5 PMC5509825

[bibr17-13623613221098240] Hull L. Petrides K. V. Mandy W. (2020). The female autism phenotype and camouflaging: A narrative review. Review Journal of Autism and Developmental Disorders, 7, 306–317.

[bibr18-13623613221098240] Hull L. Petrides K. V. Mandy W. (2021). Cognitive predictors of self-reported camouflaging in autistic adolescents. Autism Research, 14(3), 523–532. 33047869 10.1002/aur.2407

[bibr19-13623613221098240] Kenny L. Hattersley C. Molins B. Buckley C. Povey C. Pellicano E. (2016). Which terms should be used to describe autism? Perspectives from the UK autism community. Autism, 20(4), 442–462. 26134030 10.1177/1362361315588200

[bibr20-13623613221098240] Kreiser N. L. White S. W. (2014). ASD in females: Are we overstating the gender difference in diagnosis? Clinical Child and Family Psychology Review, 17(1), 67–84. 23836119 10.1007/s10567-013-0148-9

[bibr21-13623613221098240] Lai M. C. Lombardo M. V. Ruigrok A. N. Chakrabarti B. Auyeung B. Szatmari P. , . . . MRC AIMS Consortium. (2017). Quantifying and exploring camouflaging in men and women with autism. Autism, 21(6), 690–702. 27899710 10.1177/1362361316671012 PMC5536256

[bibr22-13623613221098240] Leedham A. Thompson A. R. Smith R. Freeth M. (2020). ‘I was exhausted trying to figure it out’: The experiences of females receiving an autism diagnosis in middle to late adulthood. Autism, 24(1), 135–146. 31144507 10.1177/1362361319853442

[bibr23-13623613221098240] Livingston L. A. Colvert E. , Social Relationships Study Team, Bolton P. Happé F. (2019). Good social skills despite poor theory of mind: Exploring compensation in autism spectrum disorder. Journal of Child Psychology and Psychiatry, 60(1), 102–110. 29582425 10.1111/jcpp.12886 PMC6334505

[bibr24-13623613221098240] Lockwood-Estrin G. Milner V. Spain D. Happé F. Colvert E. (2021). Barriers to autism spectrum disorder diagnosis for young women and girls: A systematic review. Review Journal of Autism and Developmental Disorders, 8, 454–470. 34868805 10.1007/s40489-020-00225-8 PMC8604819

[bibr25-13623613221098240] Loomes R. Hull L. Mandy W. P. L. (2017). What is the male-to-female ratio in autism spectrum disorder? A systematic review and meta-analysis. Journal of the American Academy of Child & Adolescent Psychiatry, 56(6), 466–474. 28545751 10.1016/j.jaac.2017.03.013

[bibr26-13623613221098240] Lyubomirsky S. Lepper H. S. (1999). A measure of subjective happiness: Preliminary reliability and construct validation. Social Indicators Research, 46(2), 137–155.

[bibr27-13623613221098240] Milner V. McIntosh H. Colvert E. Happé F. (2019). A Qualitative Exploration of the Female Experience of Autism Spectrum Disorder (ASD). Journal of Autism and Developmental Disorders, 49, 2389–2402. 30790191 10.1007/s10803-019-03906-4 PMC6546643

[bibr28-13623613221098240] Parish-Morris J. Liberman M. Y. Cieri C. Herrington J. D. Yerys B. E. Bateman L. . . . Schultz R. T. (2017). Linguistic camouflage in girls with autism spectrum disorder. Molecular Autism, 8(1), Article 48. 10.1186/s13229-017-0164-6 PMC5622482 29021889

[bibr29-13623613221098240] Russell D. W. (1996). UCLA Loneliness Scale (Version 3): Reliability, validity, and factor structure. Journal of Personality Assessment, 66(1), 20–40. 8576833 10.1207/s15327752jpa6601_2

[bibr30-13623613221098240] Rynkiewicz A. Schuller B. Marchi E. Piana S. Camurri A. Lassalle A. Baron-Cohen S. (2016). An investigation of the ‘female camouflage effect’ in autism using a computerized ADOS-2 and a test of sex/gender differences. Molecular Autism, 7(1), Article 10. 10.1186/s13229-016-0073-0 PMC4721191 26798446

[bibr31-13623613221098240] Schiltz H. K. McVey A. J. Dolan Wozniak B. Haendel A. D. Stanley R. Arias A. . . . Van Hecke A. V. (2021). The role of loneliness as a mediator between autism features and mental health among autistic young adults. Autism, 25(2), 545–555. 33126822 10.1177/1362361320967789

[bibr32-13623613221098240] Scott F. J. Baron-Cohen S. Bolton P. Brayne C. (2002). The CAST (Childhood Asperger Syndrome Test): Preliminary development of a UK screen for mainstream primary-school-age children. Autism, 6, 9–31. 11918111 10.1177/1362361302006001003

[bibr33-13623613221098240] Sedgewick F. Hill V. Yates R. Pickering L. Pellicano E. (2016). Gender differences in the social motivation and friendship experiences of autistic and non-autistic adolescents. Journal of Autism and Developmental Disorders, 46(4), 1297–1306. 26695137 10.1007/s10803-015-2669-1 PMC4786616

[bibr34-13623613221098240] Tint A. Weiss J. A. (2018). A qualitative study of the service experiences of women with autism spectrum disorder. Autism, 22(8), 928–937. 28914071 10.1177/1362361317702561

[bibr35-13623613221098240] Tubío-Fungueiriño M. Cruz S. Sampaio A. Carracedo A. Fernández-Prieto M. (2021). Social camouflaging in females with autism spectrum disorder: A systematic review. Journal of Autism and Developmental Disorders, 51(7), 2190–2199. 32926304 10.1007/s10803-020-04695-x

[bibr36-13623613221098240] The WHOQOL Group. (1998). Development of the World Health Organization WHOQOL-BREF quality of life assessment. Psychological Medicine, 28(3), 551–558. 9626712 10.1017/s0033291798006667

[bibr37-13623613221098240] Wood-Downie H. Wong B. Kovshoff H. Mandy W. Hull L. Hadwin J. A. (2021). Sex/gender differences in camouflaging in children and adolescents with autism. Journal of Autism and Developmental Disorders, 51(4), 1353–1364. 32691191 10.1007/s10803-020-04615-z PMC7985051

