# Supplementary material for: Sex differences in predictors and outcomes of camouflaging: Comparing diagnosed autistic, high autistic trait and low autistic trait young adults
Source: Autism. 2022 Jun 2;27(2):402–14. doi: 10.1177/13623613221098240 (PMC9902997; doi:10.1177/13623613221098240)
Supplement: sj-docx-1-aut-10.1177_13623613221098240 – Supplemental material for Sex differences in predictors and outcomes of camouflaging: Comparing diagnosed autistic, high autistic trait and low autistic trait young adults [file sj-docx-1-aut-10.1177_13623613221098240.docx]

**Supplementary materials:**

| **Table 4. Supplementary materials** | | | | | | | |  | | |  |  |
| --- | --- | --- | --- | --- | --- | --- | --- | --- | --- | --- | --- | --- |
|  | **Loneliness as a predictor of camouflaging for each participant group** | | | | | | | |  |  |  |  |
| **Group** | | **Predictor** | **Unstandardized Coefficient** | ***t*** | ***p*-value** | **F** | **R^2^** | | |  |  |  |
| **Dx (n=75)**  Block 1: | |  |  |  |  |  |  | | |  |  |  |
|  |  | SRS-2 score | .202 | 1.87 | .065 |  |  | | |  |  |  |
|  | | Sex | -7.13 | -1.21 | .232 | **4.18*** | **.104*** | | |  |  |  |
| Block 2: | | SRS-2 score | .098 | .885 | .379 |  |  | | |  |  |  |
|  | | Sex | -7.96 | -1.4 | .166 |  |  | | |  |  |  |
|  | | **Loneliness** | **1.46** | **2.70** | **.009** | **5.45**** | **.187**** | | |  |  |  |
| Block 3: | | SRS-2 score | .11 | 1.03 | .308 |  |  | | |  |  |  |
|  | | Sex | 19.67 | 1.06 | .295 |  |  | | |  |  |  |
|  | | **Loneliness** | **2.04** | **3.12** | **.003** |  |  | | |  |  |  |
|  | | Sex*Loneliness | -1.62 | -1.56 | .123 | **4.78**** | **.**214 | | |  |  |  |
| **HT (n=176)** | |  |  |  |  |  |  | | |  |  |  |
| Block 1: | | **SRS-2 score** | **.525** | **6.68** | **.000** |  |  | | |  |  |  |
|  | | Sex | 2.42 | .80 | .425 | **22.99***** | **.210***** | | |  |  |  |
| Block 2: | | **SRS-2 score** | **.442** | **5.45** | **.000** |  |  | | |  |  |  |
|  | | Sex | 2.21 | .75 | .456 |  |  | | |  |  |  |
|  | | **Loneliness** | **1.19** | **3.11** | **.002** | **19.32***** | **.252**** | | |  |  |  |
| Block 3: | | **SRS-2 score** | **.441** | **5.43** | **.000** |  |  | | |  |  |  |
|  | | Sex | -4.67 | -.39 | .694 |  |  | | |  |  |  |
|  | | Loneliness | .969 | 1.85 | .066 |  |  | | |  |  |  |
|  | | Sex*Loneliness | .433 | .60 | .549 | **14.53***** | .254 | | |  |  |  |
| **Comparison (n=139)** | |  |  |  |  |  |  | | |  |  |  |
| Block 1: | | **SRS-2 score** | **.61** | **5.04** | **.000** |  |  | | |  |  |  |
|  | | Sex | -1.14 | -.32 | .751 | **12.75***** | **.158***** | | |  |  |  |
| Block 2: | | **SRS-2 score** | **.504** | **4.17** | **.000** |  |  | | |  |  |  |
|  | | Sex | .346 | .10 | .922 |  |  | | |  |  |  |
|  | | **Loneliness** | **1.59** | **3.15** | **.002** | **12.35***** | **.215**** | | |  |  |  |
| Block 3: | | **SRS-2 score** | **.522** | **4.29** | **.000** |  |  | | |  |  |  |
|  | | Sex | -15.03 | -1.07 | .288 |  |  | | |  |  |  |
|  | | Loneliness | .988 | 1.34 | .183 |  |  | | |  |  |  |
|  | | Sex*Loneliness | 1.11 | 1.13 | .262 | **9.60***** | .223 | | |  |  |  |
|  | **Note:** Bold denotes predictor significant at p<.05.  *=p<0.05, ** = p<0.01, ***=p<0.001  QoL= Quality of Life; SDQ = Strengths and Difficulties Questionnaire; Sig. = Significance | | | | | | | | |  |  |  |
|  |  | | | | | | | |  |  |  |  |

| **Table 5a. Supplementary Material** | | | | | | | | |  | | |  |
| --- | --- | --- | --- | --- | --- | --- | --- | --- | --- | --- | --- | --- |
|  | **Prediction of Outcomes from Camouflaging, Sex, and Their Interaction for Diagnosed Autistic (Dx) participants** | | | | | | | | | |  |  |
| **Outcome and Model** | | **Predictor** | **Unstandardized Coefficient** | ***t*** | ***p*-value** | **Sig. After Correction** | **F** | **R^2^** | |  |  |  |
| **Subjective Happiness (n=62)** | |  |  |  |  |  |  |  | |  |  |  |
| Block 1: | | **SRS-2 score** | **-.03** | **-4.06** | **.000** | ***** |  |  | |  |  |  |
|  | | **Sex** | **-.91** | **-2.42** | **.019** | **-** | **8.58***** | **.225***** | |  |  |  |
| Block 2: | | **SRS-2 score** | **-.03** | **-3.58** | **.001** | * |  |  | |  |  |  |
|  | | **Sex** | **-.97** | **-2.63** | **.011** | * |  |  | |  |  |  |
|  | | CAT-Q total | -.01 | -1.92 | .06 | - | **7.2***** | .271 | |  |  |  |
| Block 3: | | **SRS-2 score** | **-.03** | **-3.52** | **.001** | * |  |  | |  |  |  |
|  | | Sex | -1.33 | -.89 | .377 | - |  |  | |  |  |  |
|  | | CAT-Q total | -.02 | -1.59 | .117 | - |  |  | |  |  |  |
|  | | CAT-Q * Sex | .00 | .246 | .806 | - | **5.33***** | .272 | |  |  |  |
| **Psychological QoL (n=73)** | |  |  |  |  |  |  |  | |  |  |  |
| Block 1: | | **SRS-2 score** | **-.07** | **-5.49** | **.000** | ***** |  |  | |  |  |  |
|  | | Sex | -.99 | -1.50 | .138 | - | **15.69***** | **.310***** | |  |  |  |
| Block 2: | | **SRS-2 score** | **-.06** | **-4.89** | **.000** | * |  |  | |  |  |  |
|  | | Sex | -1.15 | -1.79 | .078 | - |  |  | |  |  |  |
|  | | **CAT-Q total** | **-.03** | **-2.44** | **.017** | - | **13.18***** | .**364*** | |  |  |  |
| Block 3: | | **SRS-2 score** | **-.06** | **-4.87** | **.000** | * |  |  | |  |  |  |
|  | | Sex | -3.97 | -1.46 | .148 | - |  |  | |  |  |  |
|  | | **CAT-Q total** | **-.04** | **-2.60** | **.011** | * |  |  | |  |  |  |
|  | | CAT-Q * Sex | .03 | 1.07 | .289 | - | **10.19***** | .375 | |  |  |  |
| **SDQ Emotional Subscale (n=22)** | |  |  |  |  |  |  |  | |  |  |  |
| Block 1: | | SRS-2 score | .038 | 1.42 | .172 | - |  |  | |  |  |  |
|  | | Sex | -.742 | -.55 | .591 | - | 2.90 | .234 | |  |  |  |
| Block 2: | | SRS-2 score | .03 | .92 | .36 | - |  |  | |  |  |  |
|  | | Sex | -.90 | -.68 | .508 | - |  |  | |  |  |  |
|  | | CAT-Q total | .028 | 1.424 | .172 | - | 2.72 | .312 | |  |  |  |
| Block 3: | | SRS-2 score | .04 | 1.42 | .174 | - |  |  | |  |  |  |
|  | | Sex | 6.05 | 1.40 | .179 | - |  |  | |  |  |  |
|  | | **CAT-Q total** | **.06** | **2.24** | **.039** | ***** |  |  | |  |  |  |
|  | | CAT-Q * Sex | -.063 | -1.69 | .110 | - | 2.95 | .410 | |  |  |  |
|  | **Note:** Bold denotes predictor significant at p<.05.  * Predictor significant after correction for multiple comparisons.  QoL= Quality of Life; SDQ = Strengths and Difficulties Questionnaire; Sig. = Significance | | | | | | | | | |  |  |

| **Table 5b. Supplementary Material** | | | | | | | | | | |  |
| --- | --- | --- | --- | --- | --- | --- | --- | --- | --- | --- | --- |
|  | **Prediction of Outcomes from Camouflaging, Sex, and Their Interaction for High Autistic Trait (HT) participants** | | | | | | | | |  |  |
| **Outcome and Model** | | **Predictor** | **Unstandardized Coefficient** | ***t*** | ***p*-value** | **Sig. After Correction** | **F** | **R^2^** |  |  |  |
| **Physical QoL (n=172)** | |  |  |  |  |  |  |  |  |  |  |
| Block 1: | | **SRS-2 score** | **-.04** | **-5.11** | **.000** | ***** |  |  |  |  |  |
|  | | Sex | .21 | .629 | .530 | **-** | **13.11***** | **.134***** |  |  |  |
| Block 2: | | **SRS-2 score** | **-.04** | **-4.32** | **.000** | ***** |  |  |  |  |  |
|  | | Sex | .22 | .65 | .516 | **-** |  |  |  |  |  |
|  | | CAT-Q total | -.00 | -.389 | .698 | - | **8.74***** | .135 |  |  |  |
| Block 3: | | **SRS-2 score** | **-.044** | **-4.45** | **.000** | ***** |  |  |  |  |  |
|  | | Sex | 2.26 | 1.39 | .167 | - |  |  |  |  |  |
|  | | CAT-Q total | .00 | .43 | .667 | - |  |  |  |  |  |
|  | | CAT-Q * Sex | -.02 | -1.28 | .202 | - | **6.99***** | .143 |  |  |  |
| **Psychological QoL (n=172)** | |  |  |  |  |  |  |  |  |  |  |
| Block 1: | | **SRS-2 score** | **-.06** | **-5.40** | **.000** | ***** |  |  |  |  |  |
|  | | Sex | .40 | .996 | .321 | - | **14.82***** | **.149***** |  |  |  |
| Block 2: | | **SRS-2 score** | **-.05** | **-4.05** | **.000** | * |  |  |  |  |  |
|  | | Sex | .44 | 1.10 | .272 | - |  |  |  |  |  |
|  | | CAT-Q total | -.016 | -1.61 | .110 | - | **10.84***** | .162 |  |  |  |
| Block 3: | | **SRS-2 score** | **-.05** | **-4.15** | **.000** | * |  |  |  |  |  |
|  | | Sex | 2.58 | 1.32 | .189 | - |  |  |  |  |  |
|  | | CAT-Q total | -.01 | -.65 | .514 | - |  |  |  |  |  |
|  | | CAT-Q * Sex | -.021 | -1.12 | .266 | - | **8.45***** | .168 |  |  |  |
| **Social QoL (n=172)** | |  |  |  |  |  |  |  |  |  |  |
| Block 1: | | **SRS-2 score** | **-.03** | **-2.40** | **.018** | - |  |  |  |  |  |
|  | | Sex | -.408 | -.839 | .403 | - | **3.33*** | **.038*** |  |  |  |
| Block 2: | | SRS-2 score | -.02 | -1.07 | .286 | - |  |  |  |  |  |
|  | | Sex | -.34 | -.70 | .465 | - |  |  |  |  |  |
|  | | **CAT-Q total** | **-.028** | **-2.31** | **.022** | - | **4.05**** | **.067*** |  |  |  |
| Block 3: | | SRS-2 score | -.02 | -1.31 | .190 | - |  |  |  |  |  |
|  | | Sex | 4.37 | 1.88 | .062 | - |  |  |  |  |  |
|  | | CAT-Q total | -.01 | -.683 | .495 | - |  |  |  |  |  |
|  | | **CAT-Q * Sex** | **-.046** | **-2.07** | **.040** | - | **4.17**** | **.091*** |  |  |  |
| **Environmental QoL (n=172)** | |  |  |  |  |  |  |  |  |  |  |
| Block 1: | | **SRS-2 score** | **-.03** | **-3.35** | **.001** | * |  |  |  |  |  |
|  | | Sex | -.002 | -.004 | .996 | - | **5.63**** | **.062**** |  |  |  |
| Block 2: | | **SRS-2 score** | **-.03** | **-2.55** | **.012** | - |  |  |  |  |  |
|  | | Sex | .02 | .053 | .958 | - |  |  |  |  |  |
|  | | CAT-Q total | -.008 | -.895 | .372 | - | **4.02**** | .067 |  |  |  |
| Block 3: | | **SRS-2 score** | **-.03** | **-2.67** | **.008** | - |  |  |  |  |  |
|  | | Sex | 1.95 | 1.16 | .248 | - |  |  |  |  |  |
|  | | CAT-Q total | .000 | -.04 | .966 | - |  |  |  |  |  |
|  | | CAT-Q * Sex | -.019 | -1.17 | .243 | - | **3.36**** | .075 |  |  |  |
| **SDQ Emotional Subscale (n=30)** | |  |  |  |  |  |  |  |  |  |  |
| Block 1: | | SRS-2 score | -.017 | -.701 | .489 | - |  |  |  |  |  |
|  | | **Sex** | **-2.276** | **-2.58** | **.016** | - | **3.54*** | **.208*** |  |  |  |
| Block 2: | | SRS-2 score | -.02 | -.92 | .364 | - |  |  |  |  |  |
|  | | **Sex** | **-2.2** | **-2.45** | **.021** | - |  |  |  |  |  |
|  | | CAT-Q total | .015 | .723 | .476 | - | 2.49 | .223 |  |  |  |
| Block 3: | | SRS-2 score | -.02 | -.665 | .512 | - |  |  |  |  |  |
|  | | Sex | -5.10 | -1.08 | .291 | - |  |  |  |  |  |
|  | | CAT-Q total | .00 | .16 | .873 | - |  |  |  |  |  |
|  | | CAT-Q * Sex | .028 | .626 | .537 | - | 1.92 | .235 |  |  |  |
| **SDQ Peer Subscale (n=30)** | |  |  |  |  |  |  |  |  |  |  |
| Block 1: | | **SRS-2 score** | **.054** | **3.85** | **.001** | * |  |  |  |  |  |
|  | | **Sex** | **-1.57** | **-3.07** | **.005** | * | **12.31***** | **.477***** |  |  |  |
| Block 2: | | **SRS-2 score** | **.05** | **3.02** | **.006** | * |  |  |  |  |  |
|  | | **Sex** | **-1.48** | **2.95** | **.007** | * |  |  |  |  |  |
|  | | CAT-Q total | .018 | 1.51 | .144 | - | **9.35***** | .519 |  |  |  |
| Block 3: | | **SRS-2 score** | **.05** | **2.93** | **.007** | ***** |  |  |  |  |  |
|  | | Sex | -2.40 | -.90 | .375 | - |  |  |  |  |  |
|  | | CAT-Q total | .01 | .93 | .362 | - |  |  |  |  |  |
|  | | CAT-Q * Sex | .01 | .35 | .728 | - | **6.80***** | .521 |  |  |  |
|  | **Note:** Bold denotes predictor significant at p<.05.  * Predictor significant after correction for multiple comparisons.  QoL= Quality of Life; SDQ = Strengths and Difficulties Questionnaire; Sig. = Significance | | | | | | | | |  |  |

| **Table 5c. Supplementary Material** | | | | | | |  | |  | |
| --- | --- | --- | --- | --- | --- | --- | --- | --- | --- | --- |
| **Prediction of Outcomes from Camouflaging, Sex, and Their Interaction for comparison (not autistic) participants** | | | | | | | | | |  |
| **Outcome and Model** | **Predictor** | **Unstandardized Coefficient** | ***t*** | ***p*-value** | **Sig. After Correction** | **F** | | **R^2^** | | |
| **FQS (n= 139)** |  |  |  |  |  |  | |  | | |
| Block 1: | **SRS-2 score** | **-.01** | **-3.99** | **.000** | ***** |  | |  | | |
|  | **Sex** | **-.33** | **-4.17** | **.000** | ***** | **19.17***** | | **.220***** | | |
| Block 2: | **SRS-2 score** | **-.01** | **-3.45** | **.001** | ***** |  | |  | | |
|  | **Sex** | **-.33** | **-4.18** | **.000** | ***** |  | |  | | |
|  | CAT-Q total | -.00 | -.52 | .603 | - | **12.80***** | | .222 | | |
| Block 3: | **SRS-2 score** | **-.01** | **-3.45** | **.001** | ***** |  | |  | | |
|  | Sex | -.21 | -.62 | .538 | - |  | |  | | |
|  | **CAT-Q total** | -.00 | -.29 | .774 | - |  | |  | | |
|  | **CAT-Q * Sex** | -.00 | -.37 | .714 | - | **9.58***** | | .222 | | |
| **Physical QoL (n=128)** |  |  |  |  |  |  | |  | | |
| Block 1: | **SRS-2 score** | **-.035** | **-2.57** | **.011** | ***** |  | |  | | |
|  | Sex | .232 | .578 | .564 | **-** | **3.32*** | | **0.51*** | | |
| Block 2: | SRS-2 score | -.03 | -1.92 | .057 | **-** |  | |  | | |
|  | Sex | -.22 | .55 | .584 | **-** |  | |  | | |
|  | CAT-Q total | -.01 | -.99 | .324 | - | 2.54 | | .058 | | |
| Block 3: | SRS-2 score | -.03 | -1.92 | .058 | - |  | |  | | |
|  | Sex | -.85 | -.50 | .618 | - |  | |  | | |
|  | CAT-Q total | -.01 | -1.17 | .243 | - |  | |  | | |
|  | CAT-Q * Sex | .013 | .650 | .517 | - | 2.00 | | .061 | | |
| **Psychological QoL (n=127)** |  |  |  |  |  |  | |  | | |
| Block 1: | **SRS-2 score** | **-.073** | **-4.20** | **.000** | ***** |  | |  | | |
|  | **Sex** | **1.129** | **2.18** | **.031** | - | **10.03***** | | **.139***** | | |
| Block 2: | **SRS-2 score** | **-.06** | **-3.06** | **.003** | * |  | |  | | |
|  | **Sex** | **1.10** | **2.15** | **.034** | - |  | |  | | |
|  | CAT-Q total | -.023 | -1.88 | .063 | - | **7.99***** | | .163 | | |
| Block 3: | **SRS-2 score** | **-.06** | **-3.05** | **.003** | ***** |  | |  | | |
|  | Sex | 2.07 | .97 | .334 | - |  | |  | | |
|  | CAT-Q total | -.02 | -1.46 | .146 | - |  | |  | | |
|  | CAT-Q * Sex | -.012 | -.468 | .640 | - | **6.01***** | | .165 | | |
| **Social QoL (n=128)** |  |  |  |  |  |  | |  | | |
| Block 1: | **SRS-2 score** | **-.081** | **-4.10** | **.000** | * | **9.55***** | | **.133***** | | |
|  | Sex | -.501 | -.864 | .389 | - |  | |  | | |
| Block 2: | **SRS-2 score** | **-.06** | **-2.88** | **.005** | * |  | |  | | |
|  | Sex | -.54 | -.94 | .350 | - |  | |  | | |
|  | **CAT-Q total** | **-.029** | **-2.128** | **.035** | - | **8.06***** | | **.163*** | | |
| Block 3: | **SRS-2 score** | **-.06** | **-2.92** | **.004** | * |  | |  | | |
|  | Sex | 4.41 | 1.86 | .065 | - |  | |  | | |
|  | CAT-Q total | -.02 | -.97 | .333 | - |  | |  | | |
|  | **CAT-Q * Sex** | **-.063** | **-2.153** | **.033** | - | **7.38***** | | **.194*** | | |
| **Note:** Bold denotes predictor significant at p<.05.  * Predictor significant after correction for multiple comparisons.  FQS = Friendship Quality Scale; QoL= Quality of Life. Sig. = Significance | | | | | | | | | |  |
